# Quality Assurance of a Hospital-Based Auditory Verbal Intervention for Children with Hearing Loss

**DOI:** 10.3390/jcm14041098

**Published:** 2025-02-08

**Authors:** Signe Wischmann, Cecilia Fernandez Samar, Marianne Kyhne Hestbæk, Stefania Serafin, Per Cayé-Thomasen, Lone Percy-Smith

**Affiliations:** 1 Copenhagen Hearing and Balance Centre, Ear, Nose and Throat (ENT) and Audiology Clinic, Rigshospitalet, Inge Lehmanns Vej 8, 2100 Copenhagen, Denmark; cecilia.fernandez.samar@regionh.dk (C.F.S.); marianne.kyhne.hestbaek@regionh.dk (M.K.H.); per.caye-thomasen.01@regionh.dk (P.C.-T.); lone.percy-smith@regionh.dk (L.P.-S.); 2 Multisensory Experience Lab, Aalborg University Copenhagen, A.C. Meyers Vænge 15, 2450 Copenhagen, Denmark; sts@create.aau.dk

**Keywords:** hearing loss, AV intervention, auditory verbal therapy, implementation, hospital setting, behaviour and emotion, Strengths and Difficulties Questionnaire, children, receptive vocabulary, playful

## Abstract

**Background:** Auditory Verbal Therapy (AVT) has spread globally in the past few years due to its proven positive effect as a rehabilitation method for children with hearing loss (HL). In 2023, a three-year hospital-based AV intervention was implemented in Denmark as a government-funded offer to all children with HL. In the present study, we introduce and study a protocol for facilitating this implementation with a specific focus on clinical applicability and quality assurance. **Methods:** A working group was set up to drive the implementation process and establish a base for clinical collaboration and coproduction. The final protocol included (1) regular workshops and meetings with all AV specialists, (2) the creation of a database to collect data on the effect of the intervention, and (3) mandatory testing of the auditory skills and receptive vocabulary of the children with HL and a screening of their behavioural and emotional status with the Strengths and Difficulties Questionnaire (SDQ). **Results:** Data from 53 children with a mean age of 6.6 years were analysed in this study. Results from the SDQ showed that the children scored like the Danish norm on both the SDQ total difficulty score and SDQ prosocial score. **Conclusions:** This study’s findings indicate that children with HL, who participated in three years of hospital-based AV intervention, demonstrate the same emotional and behavioural problems and the same social strengths as their hearing peers. We hope that this study can inspire and guide others who want to implement an AV intervention in a hospital setting.

## 1. Introduction

Hearing loss (HL) can significantly impact a person’s quality of life, leading to social isolation, depression, and cognitive decline, beyond just making it difficult to hear [1]. However, over the past 30 years, a paradigm shift in audiology and hearing care has taken place. Children born deaf or with HL today have different needs and possibilities than former generations [2,3,4].

Previously, children with HL lagged significantly behind their hearing peers in terms of spoken language abilities, academic achievements, and social well-being [4]. Today, the new generation of children with HL has closed the language gap and develop age-equivalent language before entering school [5,6].

The prerequisite for this positive change is a combination of advances in several areas. The introduction of universal neonatal hearing screening (UNHS) has led to earlier identification of HL, leading to early diagnosis and intervention for children with congenital HL [4]. Technological advances and the increased accessibility of cochlear implantation have improved children’s access to sound and enabled auditory learning [7,8]. And ultimately, highly specialised rehabilitation focusing on auditory learning has advanced the development of listening and spoken language [9].

### 1.1. Rehabilitation

The foundation of auditory learning for children with HL is neuroplasticity. The neurological pathways in children born with HL have been partly or completely out of use before fitting with hearing technology [10,11,12,13]. Hence, to prevent cortical reorganisation and obtain the optimal conditions for auditory and spoken language development, intense auditory stimulation of the neurological pathways is essential [7,12]. From birth to 3½ years of age, the central auditory system remains maximally plastic, and therefore, it is important to provide acoustic and/or electrical stimulation with hearing technology (hearing aids (HAs) and/or cochlear implants (CIs)) and start auditory rehabilitation as early as possible [10,11,13].

One rehabilitation method that has spread globally for children with HL is Auditory Verbal Therapy (AVT) [14,15,16]. AVT focuses on stimulating the auditory centres of the brain, so the child learns to make sense of the sound relayed by the hearing technology [17]. The approach of the intervention is family-centred, in line with recommendations from the World Health Organization (WHO), emphasising the participation of parents and families as a key factor for optimal rehabilitation outcomes for children with HL [18]. In AVT, parents or caregivers learn to use auditory verbal (AV) strategies and techniques in everyday activities to develop the child’s neurological pathways and support listening and spoken language development [17]. As 95% of children with HL are born into families with typical hearing, most parents have a strong incentive to engage in an intervention that specifically advances this development [8,19]. Sharing the language of your hearth is fundamental for interaction between parent and child.

AVT is provided by listening and spoken language specialists (LSLS Cert AVT) who have undertaken three to five years of postgraduate training. AVT is diagnostic in its approach, meaning that it is continually tailored to the family based on formal and informal assessments that allow for the progress, or lack of progress, to be monitored and evaluated in a way that is meaningful for the family [17]. In close collaboration with the parents, the AVT specialist monitors the development of the child and sets goals for audition, speech, language, cognition, and social communication [17,20].

Several studies on children with HL, after conducting AVT, have reported positive outcomes in areas such as receptive and expressive language [3,5,9], receptive vocabulary [5,9,21,22], expressive vocabulary [9,21], reading [21], speech intelligibility [21], social skills/pragmatics [3,23,24,25], speech production [5], self-esteem [25,26], social well-being/quality of life [2,3,24], and executive functions including working memory [3,27]. In two recently published systematic reviews addressing the effect of AVT, the authors concluded that even though further well-controlled studies evaluating the use of AVT are needed, the approach is effective and ideal for children with HL [16,28]. Furthermore, auditory and language skills cannot stand alone in a holistic evaluation of the child; therefore, any paediatric intervention must include an evaluation of the social well-being level [29].

### 1.2. AVT in Denmark

In Denmark, AVT has gained ground in recent years. The starting point was the results from the first Danish outcome studies of children with CIs, which showed that the medical–surgical and technical treatment could not stand alone [25,30,31]. UNHS enabled early diagnosis and helped reduce the age of implantation, but it did not significantly change the auditory and spoken language performance of the children [30]. The Danish results were in sharp contrast to results from countries such as England, Australia, Germany, and Canada [5,8,11,32], and it became clear that guidelines for assessment, surgery, and particularly the rehabilitation of children with CIs were necessary to improve outcomes.

In 2013, two paediatric CI centres in Denmark, together with a patient organisation, initiated a partnership project. In the project, children with CIs and their families were provided with three years of AVT from speech and language pathologists who had completed three years of postgraduate AV education. The results from this project were unequivocal. Children with CIs participating in the AV intervention scored significantly higher in tests on vocabulary and language comprehension compared to children with CIs who received standard audiology/speech and language intervention. In the standard intervention, use of the method differed. It was provided by the local speech and language pathologists in the municipality, and most children received 1–2 weekly hours of speech and hearing therapy, the majority without parental participation [9].

The results from the project highlighted the importance of family involvement and quality assurance of rehabilitation and led to a national, government-funded project “The AVT-project” in 2017.

#### The AVT-Project

The AVT-project was a new larger-scale project involving the two paediatric CI centres, the same patient organisation, and a third specialised audiological hospital unit [33]. The aim of the project was to develop and test a new national setup for rehabilitation for children with all kinds of hearing technology. In the project, children aged 0–6 years with bilateral HL > 40 dB HL were offered three years of AV intervention at the audiological departments/CI centres at the hospitals. The patient organisation was responsible for setting up the framework for the project in collaboration with the hospitals, and for monitoring and documenting the outcomes of the rehabilitation. As quality assurance, nine overall goals were set up before initiating the project, comprising goals for the completion rate of the AV intervention, language acquisition, and parental satisfaction with the intervention. The project was evaluated to be a great success. During the project period, 360 children completed the three-year AV intervention, and 84% of them achieved age-equivalent language. Previously, only 30% of children with HL achieved age-equivalent language. Through the process, almost all parents (93%) were satisfied or very satisfied with the intervention, and 87% experienced increased confidence in their own abilities to support their child’s language development [33]. In addition, providing AV intervention in a hospital setting enabled a closer collaboration between the different stakeholders around the child, since speech and language pathologists/AVT specialists, audiologists, technicians, and medical doctors were physically located side by side.

In 2023, the positive evaluation of the project led to a national implementation of three years of hospital-based AV intervention as a government-funded offer to all children aged 0–6 years with uni- or bilateral HL regardless of the type of hearing technology and hearing configuration.

### 1.3. Aim

The primary aim of the present study was to introduce and study a test protocol that ensures the ongoing quality of the AV intervention. The following research questions were addressed:

- Which precursors are crucial for the successful implementation of AV intervention in a hospital setting?
- What is the behavioural and emotional status of children with HL who have participated in the three-year AV intervention in a hospital setting?

These were studied by applying a participatory design with the involvement of relevant clinical stakeholders and implementation of critical quality parameters, including social well-being.

## 2. Materials and Methods

### 2.1. Procedure for Long-Term Implementation of AVT in a Hospital Setting

**Figure 1:**
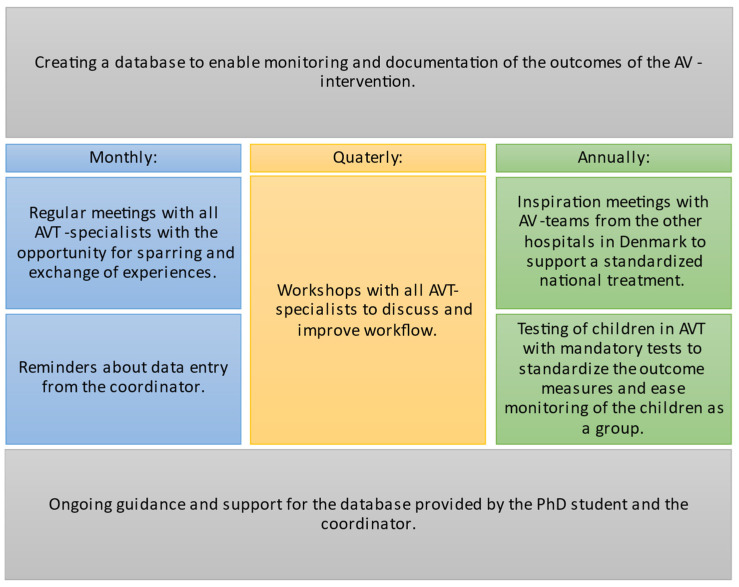
Guidelines for the final protocol.

The study was carried out at the Copenhagen Hearing and Balance Centre (CHBC) at Copenhagen University Hospital, Rigshospitalet. As the centre had been part of the AVT-project, the frame for the implementation of a permanent AV intervention, including the employment of AVT specialists, was already in place. The AVT specialists were speech and language pathologists who either trained or had already undergone special postgraduate training to become listening and language specialists. A working group was set up to facilitate implementation. It consisted of the department’s day-to-day manager, the coordinator for the AVT specialists, and a professor in speech and language pathology, all of whom were also trained AVT specialists and had participated in the AVT-project. In addition, a PhD student working with quality assurance was included to support the clinicians during the implementation process. The working group agreed on a preliminary protocol focusing on close collaboration between the AVT specialists and ensuring quality assessment of the AV intervention. The protocol was discussed with all AVT specialists at an introductory workshop and further developed over a series of iterative meetings. Guidelines from the final protocol are presented in Figure 1.

### 2.2. Quality Assurance of the AV Intervention

The AVT-project, which preceded the national hospital implementation of the intervention, documented that most children with HL achieved age-equivalent language after three years. However, a time-limited research project and a clinical routine intervention might not demonstrate the same outcomes. Hence, to ensure the continuous high quality of the AV intervention, a setup for monitoring outcomes was implemented. It was decided that all AVT specialists test the children annually using standardised tests for auditory skills and receptive vocabulary to allow comparisons between cohorts. Furthermore, there was a broad agreement that evaluations of social well-being were a fundamental aspect in all interventions involving children. According to the WHO constitution “…health is a state of complete physical, mental, and social well-being and not merely the absence of disease or infirmity” [29]. Therefore, a mandatory screening of the children’s behavioural and emotional status was introduced as a fixed outcome measure for social well-being at the end of the AV intervention. Furthermore, an important consideration was that the test protocol was clinically applicable. The tests selected for quality assurance are described in the following sections.

#### 2.2.1. Mandatory Tests

##### Auditory Skills

To assess the children’s auditory skills, parents were asked to complete the LittlEARS Auditory Questionnaire (LEAQ) [34]. The questionnaire targets children up to 2 years of age, or with a hearing age of 2 years. It includes 35 age-dependent questions to be answered with a yes or no. The questions reflect key milestones of auditory development and are based on caregivers’ observation of their child’s auditory behaviour in daily life. The total score can be used (1) to compare with previous scores to monitor the child’s progress, (2) to compare with hearing age, or (3) to compare with age-matched hearing peers.

##### Receptive Vocabulary

A Danish translation of the Peabody Picture Vocabulary Test (PPVT-4) [35] was chosen to assess receptive vocabulary. Vocabulary is crucial to monitor, as early childhood vocabulary significantly impacts academic outcomes [36].

When measured over the full range, the test targets all ages from 2½ years of age. It consists of 19 blocks of 12 tasks. In each task, the child is asked to point to one out of four pictures. The blocks are of increasing difficulty, and only those blocks that correspond to the child’s current vocabulary are administered. The number of correct answers is converted to a standard score with a mean of 100 and a standard deviation of 15. The test is norm-referenced and standardised on data from approximately 3500 subjects from the US.

##### Behavioural and Emotional Status

The Strengths and Difficulties Questionnaire (SDQ) [37] was used to assess the behavioural and emotional status of the children. The Danish version of the SDQ exists in four versions: (1) for children aged 2–4 years in nursery school, (2) for children aged 5–6 years in nursery school, (3) for children aged 4–10 years in school, and (4) for children aged 11–17 years in school. The first three versions are completed by parents and/or professionals, while the last one can also be completed by the children themselves. The questionnaire includes 25 questions to be answered with “Not true”, “Somewhat true”, or “Certainly true”. Five domains are covered in the questionnaire: emotional symptoms, conduct problems, hyperactivity/inattention, peer relationship problems and prosocial behaviour. Scores from the first four domains can be analysed separately or summed up to make a “total difficulty score”. Higher scores indicate greater difficulties. Scores from the fifth domain of “prosocial behaviour” are analysed in reverse, where higher scores reflect greater strengths. In the Danish norm, the score is divided into four categories, close to average, slightly raised/lowered, high/low, or very high/low scores with a distribution of 80%, 10%, 5%, and 5% in the respective groups [38]. The norm is based on responses from a total of 3998 parents, 5651 professionals, and 1609 children.

#### 2.2.2. Database

A database was created to gather the data collected from children participating in the AV intervention. Data were administered with the secure web application Research Electronic Data Capture System (REDCap) [39,40]. The content of the database was decided in collaboration with the working group and the AVT specialists. It included the results from the mandatory tests: LEAQ, PPVT-4, and SDQ. But there was also the possibility to enter results from optional tests carried out by the AVT specialists as part of the intervention. Optional tests included the Viborg Materialet (a Danish test of the level of active vocabulary) [41], the Reynell—Developmental Language Scales edition III [40], the New Reynell Developmental Scales (NRDLS) [42], and the Clinical Evaluation of Language Fundamentals-4 (CELF-4) [43]. In addition to the test results, the database included information on the children’s characteristics, i.e., age, gender, degree of HL, hearing technology, aetiology, and if the child had an additional disability that could have a direct impact on audition or language development. Data were entered throughout the AV intervention by the AVT specialist who was responsible for the individual child. Participation was voluntary, and informed consent was attained from the child’s family or legal caregiver before entering data about a child.

### 2.3. Social Well-Being

The systematic assessment of the behavioural and emotional status of children with HL as a part of the AV intervention was a new initiative to ensure the high quality of the programme. The results from the first study are presented in this publication.

The data are based on responses from the SDQ from children who on 1 October 2024 had completed three years of AV intervention and were still receiving treatment with hearing technology at CHBC. Both children who were already included in the database and children who had completed the AV intervention just before the database was created were invited to participate in the study. Since some of the questions in the SDQ required the child to interact with other children, children who had multiple disabilities that prevented them from being around other children were excluded from the study. A total of 53 children accepted to participate in the study. Thirty-one (58%) of the participants were girls and twenty-two (42%) were boys. The children were born from 2012 to 2020 and had a mean age of 6,6 years (SD = 2.09). Forty-six (87%) of the children were diagnosed through UNHS. Table 1 summarises the characteristics of the participants.

Parents of children aged 3–10 years completed the parent version (*n* = 50) of the SDQ, while children aged 11–17 completed the self-reported version (*n* = 3). The questionnaire was filled by pen and paper or in on electronic version sent via REDCap. The scoring of the questionnaire was performed by the AVT specialists or the PhD student.

### 2.4. Data Analysis

Descriptive statistics were summarised as frequencies and percentages. The SDQ was scored according to the Danish norm by type of respondent (parent or self-completion), age, and unisex. To compare the norm and the study group, the SDQ scores were analysed using the “N-1” Chi-squared test. *p*-values below 0.05 were considered statistically significant. A one sample *t*-test was conducted to compare the PPVT-4 mean score with the norm, and finally, Spearman’s correlation coefficient was used to test for correlation between the SDQ and PPVT-4 scores.

## 3. Results

Table 2 summarises the descriptive statistics for outcomes on the SDQ.

All SDQ scores showed a distribution like that of the Danish norm. For the total difficulty score, 43 children (81%) fell in the “close to average” category, 8 children (15%) in the “slightly raised” category, 1 child (2%) fell in the “high” category, and another child (2%) in the “very high” category. For prosocial behaviour, 46 children (87%) scored “close to average”, 2 (4%) scored “slightly lowered”, 3 (6%) scored “low”, and 2 children (4%) scored “very low”. N-1 Chi-squared tests indicated that there were no significant differences between the children with HL and the Danish norm on either of the scores.

Data on PPVT-4 followed a normal distribution, and a one-sample *t*-test was conducted to compare the average score on PPVT-4 against the norm of 100. The results indicate that the mean standard score (M = 96, SD = 22.61) was not significantly different from the norm: t(48) = 1.238, *p* = 0.222. <0.05.

**Figure 2:**
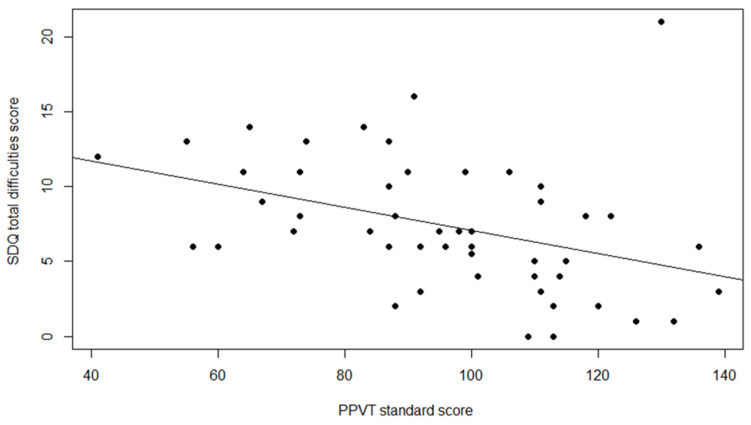
Relation between PPVT-4 standard score and SDQ total difficulty score.

Finally, Spearman’s correlation coefficient was computed to determine the relationship between the PPVT-4 standard score and SDQ total difficulties score. Spearman’s correlation was used since the data on the SDQ did not follow a normal a distribution. PPVT-4 data were missing for 4 children, so only 49 children were included in the analyses. The results show a moderate negative correlation between the two variables rho = −0.50 and *p* = 0.000, indicating that higher (better) scores on PPVT-4 were associated with lower (better) scores on the SDQ. Figure 2 displays the relation between the PPVT-4 standard score and SDQ total difficulty score in a scatterplot. No significant correlations were found between the PPVT-4 and prosocial score.

## 4. Discussion

A range of evidence shows the benefits of AVT, and every year, more research is published [17,44]. However, the provision of knowledge on its own is not enough to change clinical practice [45]. In the present study, we introduced and studied a test protocol to facilitate the implementation and quality assurance of a hospital-based AV intervention. It must be emphasised that although the AV intervention was implemented nationally, the protocol and results presented in this study are only valid for CHBC at Copenhagen University Hospital, Rigshospitalet. In the future, it would be appropriate to unify the quality standards on all hospitals providing the AV intervention to ensure equality in healthcare for all children in Denmark. However, implementation at a national level may be challenging as the five regions each have their own database system.

In drawing on knowledge from implementation science, a working group with strong facilitative leaders was set up to drive the implementation process and establish a base for clinical collaboration and coproduction [46]. This setup proved effective in initiating the development of a protocol that was clinically applicable. The final protocol included regular workshops and meetings focusing on audit and feedback to emphasise collaboration, collective action, and reflexive monitoring [47]. Furthermore, the protocol included the mandatory annual testing of children with HL and the creation of a database to enable the documentation of the effects of the AV intervention. In the future, it may be relevant to include information on novel biomarkers and factors implicated for auditory disorders [1]. Parents’ demand for information regarding the genetics of hearing loss will also be addressed by future stem cell treatments for hearing loss.

It can be discussed whether creating a new database to collect information on the children’s characteristics and test results is the most appropriate solution for documenting outcomes. Medical information collected about patients at hospitals in the Capital Region in Denmark, including test results from AV intervention, is always registered in their online medical records on “the Healthcare Platform”. Through the creation of a new database, responsibility is placed on the AVT specialists to enter the same data in two different platforms (the REDCap database and the Healthcare Platform). This is time-consuming and might lead to oversights and missing data, which compromises quality. Moreover, registration in the REDCap database requires separate and annual informed consent from the child’s family or legal caregiver, which might exclude some children from participating. Therefore, the setup for monitoring the AV intervention may be improved if the collection and comparison of test results were performed directly in the existing Healthcare Platform. The working group is currently considering potential solutions for this.

A mandatory screening of the children’s behavioural and emotional status was introduced in the present study as an outcome measure for social well-being after three-years of AV intervention. Children’s social well-being is a relevant and important topic and an area where, until recently, children with HL have faced serious challenges compared to hearing children [48,49]. However, data from this study showed positive results. Children with HL who participated in three years of hospital-based AV intervention scored like the Danish norm on both the SDQ total difficulty score and SDQ prosocial score, indicating that they demonstrate the same emotional and behavioural problems, and the same social strengths as their hearing peers.

These results are in line with other studies on the emotional and behavioural status of the new generation of children with HL. In a recent Danish study using a parent- and self-reported SDQ with 22 children with HL, the authors reported that more than 94% of the children fell in the “close to average” category on both the total difficulty score and the prosocial score. None of the children fell in the critical “high/low” or “very high/very low” categories on either of the scores [2]. In a larger-scale study on the emotional and behavioural status of 144 children with HL, Australian researchers also reported positive emotional and behavioural status with average SDQ scores within one SD of the normative data. However, in contrast to the present study, they noted that a greater proportion of children with HL scored below two SDs of the norm [24]. Interestingly, they found that functional auditory performance and pragmatic language skills were significant predictors of the SDQ scores. Since these two abilities are trained in AVT, we hypothesise that the positive SDQ scores obtained in our study will be the standard for children with HL who participate in hospital-based AV interventions in the future.

Other variables that have frequently been associated with children’s social well-being are structural language abilities [50,51,52,53]. In our study, we investigated the association between receptive language (PPVT-4) and the SDQ and found that higher receptive language was related to better behavioural and emotional status, as measured by the SDQ total difficult score but not the SDQ prosocial score.

In addition to the total difficulty score and the prosocial score, the SDQ has an extended version that includes an “impact score”. The impact score consists of five supplementary questions, where the respondents are directly asked if they think that the child has difficulties and how these difficulties interfere with the child’s everyday life [54]. Some studies report that the impact score is more sensitive than the total problem score in capturing the seriousness of difficulties [54,55]. However, to the best of our knowledge, the impact score has not been validated or recommended as appropriate to use with children with HL, and therefore, it was not included in the present study.

The SDQ was applied in this study because the psychometric properties and user acceptability of the questionnaire as a clinical and research tool have been well established. However, applying the SDQ as a screening tool for the behavioural and emotional status of children with HL presents some limitations. The SDQ is from the end of the last millennium, and it might be time to revise the questions in terms of a more updated vocabulary targeting today’s children. In their study from 2024, Hammer et al. found that children perceived several of the questions in the SDQ as “old-fashioned” [2]. Based on conversations with the children, they concluded that words such as bully, stomach aches, headaches, and unhappy might not be appropriate as they risk defining the children’s feelings by “putting words into their mouth”.

Furthermore, the questionnaire is not suitable for all children. Some of the questions require the child to interact with other children and therefore exclude some children with severe multiple disabilities. Future studies should preferably use questionnaires that comprise all children with HL to provide knowledge regarding the entire population.

A recent paper [56] underlined the importance of continuously monitoring children’s language progress to ensure that no child is left behind in terms of acquiring a language. It is, furthermore, underlined that some children will need a complete communication approach and few children will need to acquire sign language (SL). The percentage of how many children will acquire (SL) was recently studied by Howell et al., 2024. In a cohort of 997 children with hearing loss from 2012 to 2021, 8.7% used Auslan, and of the 8.7%, only 2.6% used Auslan as their primary language at home [57].

## 5. Conclusions

In this study, we proposed a protocol for the implementation of a hospital-based AV intervention with a specific focus on clinical applicability and quality assurance. The protocol was based on a collaborative approach with regular workshops and meetings with all AVT specialists, encouraging collective action and reflexive monitoring. Annual tests were introduced, and data were collected in a database to enable the systematic and continuous monitoring and documentation of outcomes. A screening of the behavioural and emotional status of the children was included at the end of the intervention, and results from this study were presented. The results indicate that children with HL who participated in the three-year AV intervention at CHBC do not deviate from their hearing peers in terms of emotional and behavioural problems or social strengths.

This study adds to other studies reporting positive outcomes from the new generation of children with HL who have benefited from early detection, early fitting with advanced hearing technology, and highly specialised auditory verbal rehabilitation.

Children have the right to the highest attainable standards of health, including quality treatment and rehabilitation [58]. We hope that the experiences presented in this paper can inspire and guide others who want to implement an AV intervention in a hospital setting.

## Figures and Tables

**Table 1:** Characteristics of participants.

Characteristics		*n* (%)
Gender	Boy	22 (42%)
	Girl	31 (58%)
Diagnosis of HL/Aetiology ^1^	Birth complications	1 (2%)
	Infections	6 (11%)
	Auditory neuropathy	2 (4%)
	Hereditary HL	16 (30%)
	Genetics without evidence of heredity	4 (8%)
	Syndromes	17 (32%)
	Ear anomalies	5 (9%)
	Other	2 (4%)
	Unknown	17 (32%)
Additional disabilities		8 (15%)
Hearing device	Bilateral CI	25 (47%)
	Bilateral HA	15 (28%)
	Bimodal CI/HA	12 (23%)
	Bilateral BAHS	1 (2%)
Degree of hearing loss	41–60 dB HL (moderate)	11 (21%)
	61–80 dB HL (severe)	5 (9%)
	>80 dB HL (profound)	37 (70%)
Diagnosed through UNHS	Yes	46 (87%)
	No	7 (13%)
Parent–child language	Danish	36 (68%)
	Danish and/or another language ^2^	16 (30%)
	Danish/Arabic/sign-support	1 (2%)
Mean age in years (SD)	Start of AVT	1.8 (1.5)
	End of AVT	5.1 (2.1)
	Test with PPVT-4	4.9 (1.6)
	Test with SDQ	6.6 (2.1)

^1^ Children could have more than one diagnosis. ^2^ Thai, Kurdish, Urdu, French, Turkish, Arabic, Serbian, Spanish/Italian, Russian, Chinese, Turkish/Polish, English.

**Table 2:** Outcomes of SDQ (*n* = 53).

SDQ	Close to Average *n* (%)	Slightly Raised/ Slightly lowered *n* (%)	High/ Low *n* (%)	Very High/ Very Low *n* (%)
Emotional problems *	49 (92%)	3 (6%)	0 (0%)	1 (2%)
Conduct problems *	46 (87%)	4 (8%)	2 (4%)	1 (2%)
Hyperactivity *	44 (83%)	3 (6%)	2 (4%)	4 (8%)
Peer problems *	46 (87%)	1 (2%)	4 (8%)	2 (4%)
Total difficulties *	43 (81%)	8 (15%)	1 (2%)	1 (2%)
Prosocial behaviour **	46 (87%)	2 (4%)	3 (6%)	2 (4%)

* According to the Danish norm, the SDQ scores are distributed as follows. Slightly raised: At least 80% have a lower score. High: At least 90% have a lower score. Very high: At least 95% have a lower score. ** The “Prosocial behaviour” scores in the norm are distributed as follows. Slightly lowered: At least 80% have a higher score. Low: At least 90% have a higher score. Very low: At least 95% have a higher score.

## Data Availability

Data are unavailable due to privacy restrictions as participants have not given permission to share data with people not directly involved in this study.
